# Spinal Loads during Post-Operative Physiotherapeutic Exercises

**DOI:** 10.1371/journal.pone.0102005

**Published:** 2014-07-07

**Authors:** Antonius Rohlmann, Verena Schwachmeyer, Friedmar Graichen, Georg Bergmann

**Affiliations:** Julius Wolff Institute, Charité – Universitätsmedizin Berlin, Berlin, Germany; Toronto Western Hospital, Canada

## Abstract

After spinal surgery, physiotherapeutic exercises are performed to achieve a rapid return to normal life. One important aim of treatment is to regain muscle strength, but it is known that muscle forces increase the spinal loads to potentially hazardous levels. It has not yet been clarified which exercises cause high spinal forces and thus endanger the surgical outcome. The loads on vertebral body replacements were measured in 5 patients during eleven physiotherapeutic exercises, performed in the supine, prone, or lateral position or on all fours (kneeling on the hands and knees). Low resultant forces on the vertebral body replacement were measured for the following exercises: lifting one straight leg in the supine position, abduction of the leg in the lateral position, outstretching one leg in the all-fours position, and hollowing the back in the all-fours position. From the biomechanical point of view, these exercises can be performed shortly after surgery. Implant forces similar or even greater than those for walking were measured during: lifting both legs, lifting the pelvis in the supine position, outstretching one arm with or without simultaneously outstretching the contralateral leg in the all-fours position, and arching the back in the all-fours position. These exercises should not be performed shortly after spine surgery.

## Introduction

Patients with a severe compression fracture of a vertebral body are often surgically treated with an internal spinal fixation device implanted from the posterior and with a vertebral body replacement (VBR) inserted anteriorly. During the rehabilitation phase, physiotherapeutic exercises are performed to regain muscle strength and to enhance a rapid return to normal life. To improve their strength, the muscles must be activated accordingly. However, high trunk muscle forces cause high loads in the corresponding spine region, which can result in implant subsidence, pedicle screw loosening, or even implant failure. Therefore, physiotherapeutic exercises that can cause high spinal loads should be avoided, but it is unclear which exercises are most relevant.

Only a few existing musculoskeletal models [1]–[7] are appropriate for calculating the spinal loads during complex activities. Validation of these models for complex activities is difficult due to a lack of experimental data.

Intradiscal pressure has been measured *in vivo* for many different activities [8]–[11], but only a few data exist for physiotherapeutic exercises.

The loads on internal spinal fixation devices during physiotherapy were measured in 10 patients [12]. Activities while lying, sitting, standing, and kneeling on the hands and knees (all-fours position) were investigated. The highest loads were measured for walking and activities during standing. The preliminary results for the loads on a VBR, measured shortly after surgery in up to 3 patients, showed high implant forces for forward flexion of the upper body, ascension of stairs, and elevation of both arms with weights in the hands or against the resistance applied by a physiotherapist [13], [14]. The implant loads during sitting, walking, whole-body vibration, and changing body position were also measured [15]–[18]. However, little information exists regarding the loads for exercises in lying positions or the all-fours position.

The aim of this paper is to present the loads on a VBR, measured during physiotherapeutic exercises in a lying position and while kneeling on the hands and knees. The results may help physiotherapists select the optimal exercises for patients — depending on the postoperative time point — to avoid high spinal loads, which may endanger clinical outcomes.

## Materials and Methods

### Ethics statement

The Ethics Committee of Charité – Universitätsmedizin Berlin approved implantation of the telemeterized VBR in patients and the subsequent measurements (registry number 213-01/225-20). Before surgery, the procedure was explained to the patients, and they provided their written informed consent for the implantation of the modified implants and the obtaining of load measurements. Measurements were permitted within a maximum period of 6 years.

### Telemeterized VBR

The clinically used VBR Synex™ (Synthes Inc., Bettlach, Switzerland) was redesigned. Six load sensors (strain gauges), a telemetry unit, and a coil for the inductive power supply were inserted into a cylindrical tube, with the original implant endplate on one side. A plate was electron-beam-welded on the other side to close the tube. Screwed-on endplates of various heights allowed for intraoperative adaptation of the implant height to the defect length. Telemetry was only active within a magnetic field of 4 kHz. Before implantation in patients, the VBRs were calibrated extensively by applying 21 different load combinations [19]. The typical average measuring errors were less than 2% for forces and less than 5% for moments related to the maximum applied force (3000 N) and moment (20 Nm). The resolution was better than 1 N and 0.01 Nm. The instrumented implants are described in detail elsewhere [20].

### Subjects

Telemeterized VBRs were implanted in five patients (4 male, 1 female, 62–71 years old, 60–74 kg body mass, 168–180 cm height, 19–26 kg/m^2^ body mass index). Four patients (WP1–WP4) each had a compression fracture of the L1 vertebral body and one (WP5) of the L3 vertebral body. In the first step, internal spinal fixation devices, spanning 2 segments in 3 patients and 4 segments in 2 patients (WP3 and WP4), were implanted from the posterior to stabilize the spine. In a second operation, a ventrolateral approach was used to perform partial corpectomy of the fractured vertebra and to remove parts of the adjacent discs. The telemeterized VBR was inserted in the created niche. Bone material from the iliac crest and the resected rib was used to cover the implant and to enhance the interbody fusion process. The internal fixation devices remained in the patient after insertion of the VBR.

For the measurements, a power coil was placed around the patient's trunk, and a small wire antenna was placed on the patient's back; both devices were fixed with a harness [21]. The signals of telemetry were transmitted to a notebook computer, with which the loads were calculated. The patients were videotaped during the measurements, and the load-dependent signals of telemetry were stored simultaneously on the audio track of the same videotape. This process enabled a detailed analysis of implant loads later, without the patient having to be present.

### Exercises

Measurements of the VBR loads began a few days after surgery and were repeated 15–28 times at follow-up sessions over a period of up to 65 months postoperatively. Approximately 25 standard exercises, such as flexion, extension, lateral bending and axial rotation of the upper body, walking, and elevation of an arm with a weight in the hand, were performed during almost all of the 97 measuring sessions. The loads were measured during approximately 1000 different combinations of activities and parameters.

Eleven exercises performed in a lying position and the all-fours position were investigated in the present study. These exercises are described in Table 1. Only a few general instructions were given to the patients because we wanted to measure the everyday situations in which the patients would perform the exercises, without the supervision of a physiotherapist. The measurements were obtained over a time period of approximately 5 years. The number of repetitions varied greatly for the various exercises. Not all the patients agreed to perform all the exercises. In some cases, a patient was unable to perform a certain exercise due to a lack of strength.

**Table 1:** Description of the investigated exercises.

Exercise number	Body position	Exercise	Total number (range per patient) of measurements
1	Lying supine	Lifting one straight leg	94 (8 to 40)
2	Lying supine	Lifting both straight legs	41 (1 to 21)
3	Lying supine	Lifting the pelvis (Bridging)	93 (10 to 32)
4	Lying prone	Lifting one cranially stretched arm	62 (6 to 33)
5	Lying prone	Lifting one arm and the contralateral leg	44 (6 to 23
6	Lying lateral	Abduction of a straight leg	64 (2 to 33)
7	All-fours position	Outstretching one arm cranially	15 (2 to 7)
8	All-fours position	Outstretching one leg	8 (2 to 2)
9	All-fours position	Outstretching one arm and the leg on the contralateral side	23 (2 to 12)
10	All-fours position	Flexing the lumbar spine (Arching)	16 (2 to 7)
11	All-fours position	Extending the lumbar spine (Hollowing)	13 (2 to 5)

### Evaluation

The forces were measured throughout each exercise. The peak value during an exercise was compared to the mean value in the resting position. Hence, the increase in the resultant peak force on the implant due to exercise is reported here. The median values and ranges of the resultant force increases were determined. The resultant force is the geometrical sum of the three force components. For an ‘average patient’, the medians and the ranges of the medians for single patients are given.

Walking is the most important activity in daily life that causes higher spinal loads than lying, sitting, or standing. Loads less than those during walking are regarded as safe. Therefore, the individual resultant forces for physiotherapeutic exercises are compared with those during level walking in the same patient [22].

## Results

### Exercises in a lying position

In the lying position, the resultant force on the VBR was usually small (less than 50 N). Due to higher muscle tension, e.g., when awaiting an arduous exercise or when lying in an uncomfortable position, the force could increase to 100 N.

#### Supine position

**Figure 1:**
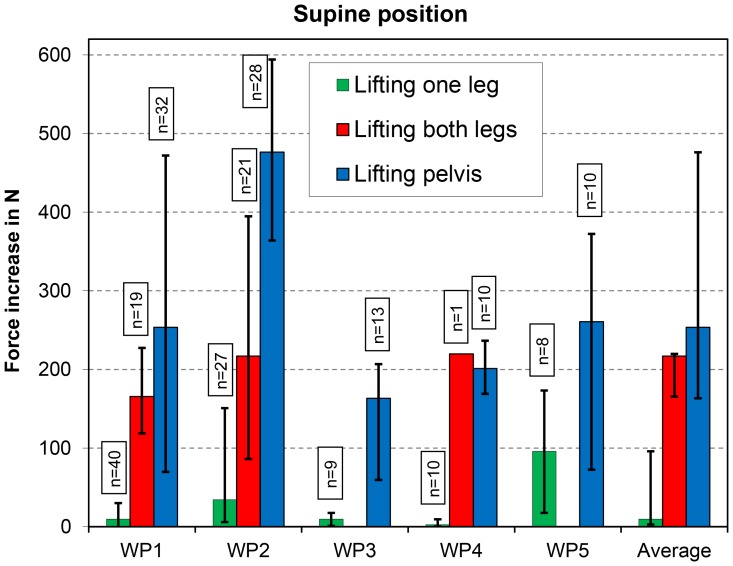
Supine position. Increases in peak force for three exercises. Data from 5 patients (WP1–WP5). The median values and ranges of the single patients and their averages are shown. n = number of evaluated trials. The force increase relates to the relaxed resting position.

Lifting a straight leg in the supine position (exercise 1 in Table 1) led to an average force increase of only 10 N (Figure 1). However, in one patient (WP5), the increase was nearly 100 N. Lifting both straight legs (exercise 2) caused a force increase of nearly 220 N. Lifting the pelvis (exercise 3) led to an average force increase of approximately 250 N. Particularly with this exercise, there was large intra-individual variation, depending strongly on the height of the pelvis lifting.

#### Prone position

**Figure 2:**
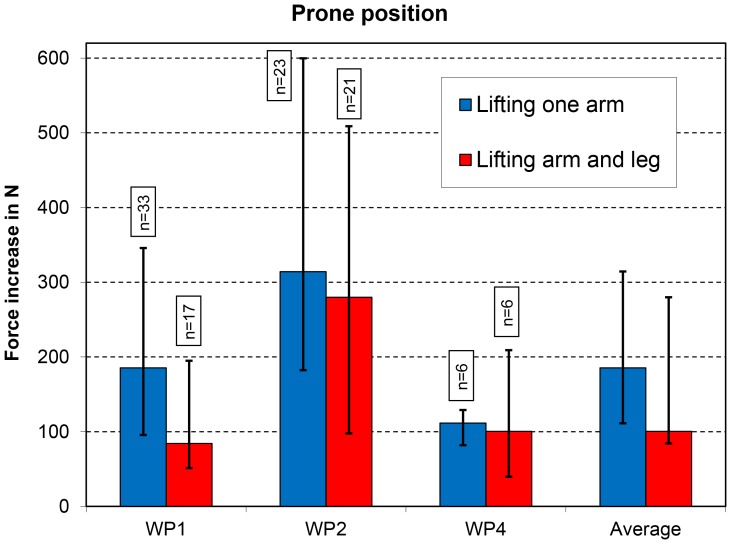
Prone position. Increases in peak force for two exercises. Data from 3 patients (WP1, WP2, and WP4). See also Figure 1.

Lifting a cranially stretched straight arm (exercise 4) increased the force on the VBR by an average of approximately 185 N (Figure 2). Additionally, with this exercise, there were large inter- and intra-individual variations in the force increases. Surprisingly, lifting an arm and the contralateral leg (exercise 5) led mostly to a smaller force increase (100 N) than only lifting an arm.

#### Lateral position

**Figure 3:**
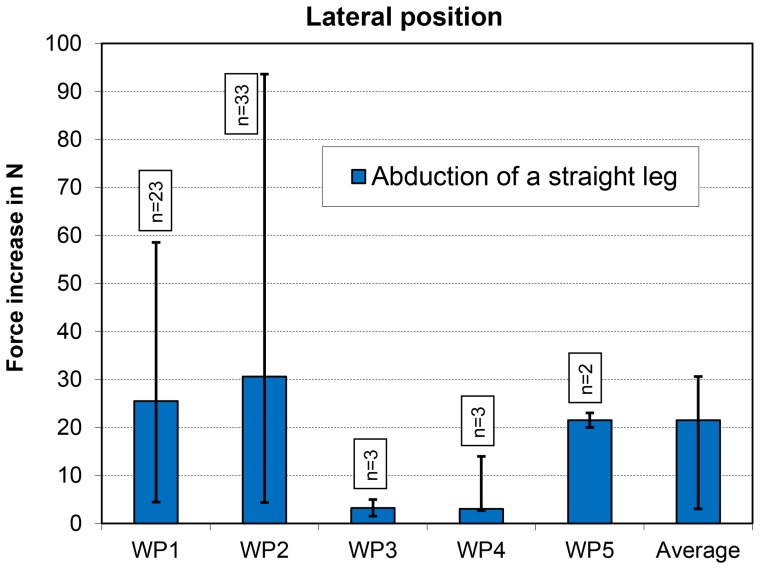
Lateral position. Increases in peak force for abduction of a straight leg. Data from 5 patients (WP1–WP5). See also Figure 1.

Abduction of a straight leg in the lateral position (exercise 6) caused a median force increase of only 22 N (Figure 3). Additionally, with this exercise, there was large intra- and inter-individual variation.

### Exercises in the all-fours position

In the relaxed all-fours position, the average resultant force on the VBR for patients WP1, WP2, WP4, and WP5 were 81 N, 169 N, 74 N, and 121 N, respectively.

#### Outstretching one arm or leg

**Figure 4:**
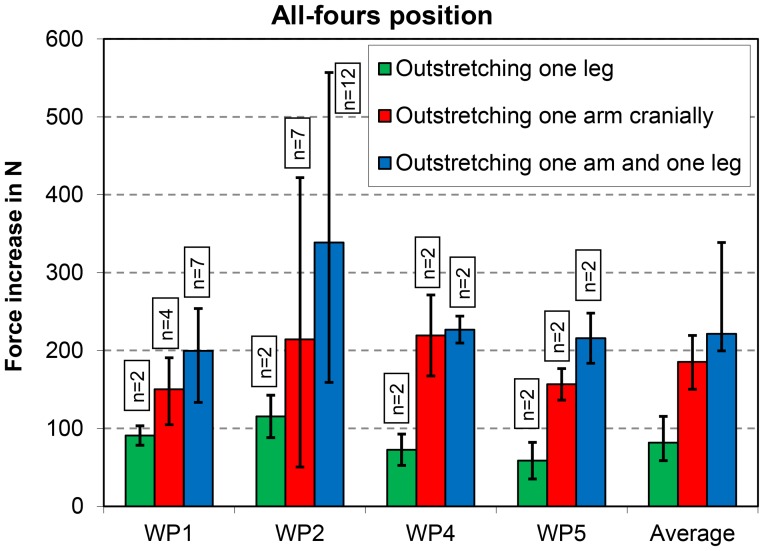
All-fours position. Increases in peak force for three exercises. Data from 4 patients (WP1, WP2, WP4, and WP5). See also Figure 1.

Outstretching one leg in the all-fours position (exercise 7) usually resulted in a force increase of less than 100 N, while outstretching one arm cranially (exercise 8) increased the force on the VBR by approximately twice that value (Figure 4). Outstretching one arm and the contralateral leg (exercise 9) caused a median force increase of 220 N, but in one patient, the maximum force increase was 555 N.

#### Arching and hollowing the back

**Figure 5:**
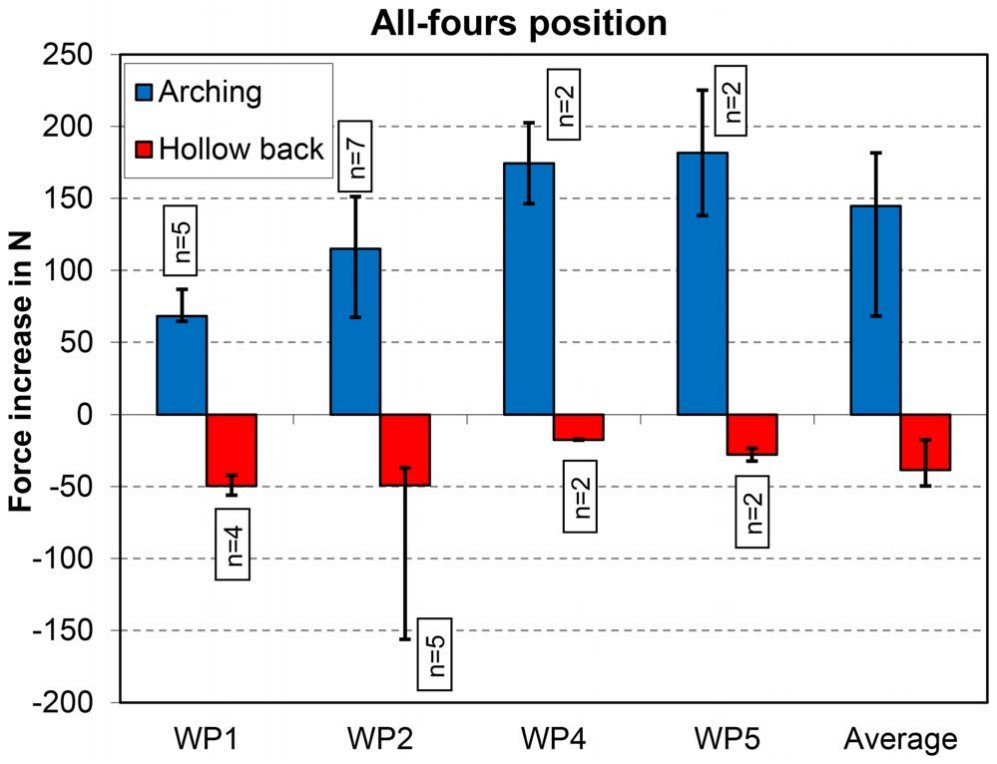
Changing spinal shape. Increases in peak force for arching and hollowing the back in the all-fours position. Data from 4 patients (WP1, WP2, WP4, and WP5). See also Figure 1.

Arching the back in the all-fours position (exercise 10) led to a median force increase of approximately 150 N (Figure 5). In contrast, hollowing the back (exercise 11) reduced the resultant force on the VBR by approximately 40 N. Additionally, with the exercises in the all-fours position, the force increases varied greatly both intra- and inter-individually.

## Discussion

The resultant forces on a VBR were measured for physiotherapeutic exercises in three different lying positions and the all-fours position. Median force increases of more than 200 N were measured for lifting both legs, lifting the pelvis in the supine position, and outstretching one arm and the contralateral leg in the all-fours position.

The average force increases for exercises in a lying position were less than 25 N for lifting one leg in the supine position and for abducting one leg in the lateral position. Therefore, from a biomechanical point of view, these exercises can be performed shortly after spine surgery. Lifting one cranially stretched arm and the contralateral leg in the prone position caused a median force increase of 100 N and is also an exercise that can be performed shortly after surgery. However, lifting both extended legs and lifting the pelvis in the supine position caused median force increases of more than 200 N. These exercises create a resultant force that might be even greater than that during walking [15], [22]. Walking is considered to be the most important activity with relatively high loads that everyone should perform daily. Therefore, these physiotherapeutic exercises should not be performed shortly after spine surgery or when patients have back problems.

Outstretching one leg in the all-fours position usually increased the force on the VBR by less than 100 N. Thus, this exercise should not place the patient at risk. Outstretching an arm cranially, with or without simultaneous outstretching of the leg, led to a force increase of approximately 200 N and thus to a force on the VBR that might be greater than that during walking [15], [22]. Therefore, these exercises should not be performed shortly after spine surgery. Arching the back in the all-fours position also led to resultant forces on the VBR that were similar to those during walking. In contrast, hollowing the back reduced the forces on the VBR but most likely increased the compressive forces on the facet joints. If the facet joints generate pain, then this exercise should not be performed.

For nearly all the activities studied, there were large intra- and inter-individual variations in the resultant force increases. The variations might have been due to small deviations in the surgical procedures, different performances of the exercises, different postoperative times, subsidence of the VBR, differences in body weight, and different positions of the center of mass of the upper body due to various factors, e.g., different positions of the head or of the extremities [15], [16], [22], [23]. The manner in which the exercises were performed varied because only a few general instructions were given to the patients. Physiotherapists can influence loads by providing specific instructions. Particularly in lifting the pelvis, the lifting height had a strong influence on the force increase. Therefore, lifting the pelvis *not* up to the maximum possible level may help to avoid high spinal loads. However, the results presented here describe the spinal loads during patient training in daily life.

The following limitations should be borne in mind. The spinal load at the implant level was shared by the VBR, the internal fixation device, the remaining bone, and the added bone material. The instrumented VBR measured only the loads on the spinal implant and not the complete loads across the entire segment. Measurements could be obtained only in a small cohort of 5 patients, making it difficult to draw general conclusions from the results, particularly when they varied greatly among the subjects. The numbers of trials and the postoperative times varied among the various patients. These patients were involved in several other load-measuring studies [15], [22]–[24]. To avoid overstressing the patients, the numbers of activities and repetitions were limited. This limitation and the large number of different exercises studied did not allow for motion analyses to be performed or for other additional parameters to be measured. Despite these limitations, this study still provided novel, unique, and helpful information regarding the loads acting on VBRs during physiotherapeutic exercises. This information may help physiotherapists to choose the most appropriate postoperative exercises for an individual patient.

In summary, the resultant forces on a VBR were measured for eleven physiotherapeutic exercises performed in lying positions or the all-fours position. Forces similar to or even greater than those for walking [15], [22] were measured for lifting both legs, lifting the pelvis in the supine position, outstretching one arm with or without simultaneous outstretching of the contralateral leg in the all-fours position, and arching the back in the all-fours position. These exercises should not be performed shortly after spine surgery.
